# Machine learning to predict mortality for aneurysmal subarachnoid hemorrhage (aSAH) using a large nationwide EHR database

**DOI:** 10.1371/journal.pdig.0000400

**Published:** 2023-12-06

**Authors:** Gen Zhu, Anthony Yuan, Duo Yu, Alicia Zha, Hulin Wu

**Affiliations:** 1 Global Health & Analytics, Development, Novartis Pharmaceuticals, East Hanover, New Jersey, United States of America; 2 Department of Internal Medicine, The University of Texas Southwestern, Texas, United States of America; 3 Division of Biostatistics, Institute for Health & Equity, Medical College of Wisconsin, Milwaukee, Wisconsin, United States of America; 4 Department of Neurology, The Ohio State University Wexner Medical Center, Columbus, Ohio, United States of America; 5 Department of Biostatistics and Data Science, The University of Texas Health Science Center at Houston, Houston, Texas, United States of America; CSL Behring / Swiss Institute for Translational and Entrepreneurial Medicine (SITEM), SWITZERLAND

## Abstract

Aneurysmal subarachnoid hemorrhage (aSAH) develops quickly once it occurs and threatens the life of patients. We aimed to use machine learning to predict mortality for SAH patients at an early stage which can help doctors make clinical decisions. In our study, we applied different machine learning methods to an aSAH cohort extracted from a national EHR database, the Cerner Health Facts EHR database (2000–2018). The outcome of interest was in-hospital mortality, as either passing away while still in the hospital or being discharged to hospice care. Machine learning-based models were primarily evaluated by the area under the receiver operating characteristic curve (AUC). The population size of the SAH cohort was 6728. The machine learning methods achieved an average of AUCs of 0.805 for predicting mortality with only the initial 24 hours’ EHR data. Without losing the prediction power, we used the logistic regression to identify 42 risk factors, —examples include age and serum glucose—that exhibit a significant correlation with the mortality of aSAH patients. Our study illustrates the potential of utilizing machine learning techniques as a practical prognostic tool for predicting aSAH mortality at the bedside.

## Author summary

The enormous amount of data available in the EHR offers an excellent environment for applying and developing machine learning and artificial intelligence (AI) methods to solve clinical research problems. In our study, we aim to use machine learning and full clinical information to predict in-hospital mortality for an aSAH cohort obtained from a national EHR database. For aSAH which is a severe type of stroke, most studies used variables such as age, size and location of the ruptured aneurysm to predict the death. They did not consider machine learning methods as the prediction tools and fully utilize the available clinical variables which can be over 1,000. We will show how modern machine learning methods can be employed to conduct the bedside prediction for mortality after aSAH.

## Introduction

### EHR and machine learning

The enormous amount of data available in the EHR offers a great environment for applying and developing machine learning and artificial intelligence (AI) methods, to address clinical research challenges. Unlike traditional cohort studies that are designed to follow people for years, EHR offers access to a wealth of data from more time points and larger populations at a lower cost. Additionally, contemporary statistical and machine learning models are increasingly being leveraged to support clinicians in making informed clinical decisions using EHR data [1,2]. For example, there is a growing body of research employing machine learning methods and EHR data for risk prediction [3]. Various deep learning techniques have been employed for tasks such as disease classification and the sequential prediction of clinical events from EHR data [4]. Notably, machine learning has demonstrated promising results in predicting mortality for various diseases. For example, a random forest model achieved an Area Under the Receiver Operating Characteristic Curve (AUC) of 0.86 in predicting in-hospital death for sepsis patients [5]. An exhaustive review of machine learning-based mortality prediction for cancer patients using EHRs has been conducted [6]. For acute diseases such as stroke, the ability of machine learning to predict the likelihood of death can aid physicians in optimizing treatment plans and making more evidence-based decisions promptly. In this study, we investigated the use of machine learning to predict mortality for aneurysmal SAH (aSAH) based on a comprehensive national EHR database.

### Mortality prediction for aSAH

aSAH is a neurologic emergency characterized by the extravasation of blood into the spaces covering the central nervous system that are filled with cerebrospinal fluid. The leading cause of nontraumatic subarachnoid hemorrhage is rupture of an intracranial aneurysm [7]. The reported incidence of aSAH is 2–22.5 cases per 100, 000 persons [8] with a high rate of fatality (25–50%) and a severe loss of productive life [8,9]. Surgical clipping or endovascular coiling of the ruptured aneurysm are the common surgical treatments for aSAH. Complications of SAH include vasospasm, delayed cerebral ischemia and hydrocephalus. Medications such as nimodipine are commonly used to reduce the risk of complications such as cerebral vasospasm [8,10,11].

Prior research has explored the utilization of machine learning techniques for predicting various outcomes, including delayed cerebral ischemia and Glasgow Coma Scale scores, using retrospective hospital data [12,13]. However, studies focused on predicting mortality in the context of SAH have been relatively scarce, primarily relying on clinical trial data [14]. Most of these studies have tended to concentrate on well-known predictors of outcomes, such as age, clinical severity scales, clipping, and coiling, which do not fully leverage the wealth of available patient information. Consequently, their predictive power has been modest, typically yielding an AUC of 0.81 [15,16]. In one study, XGBoost was employed to forecast mortality within a cohort of 351 patients from a single hospital, achieving an impressive AUC of 0.95 [17]. However, the notable performance of this model in a singular hospital setting may not necessarily extend to other data sources.

Authors have recently applied machine learning techniques to a cohort of patients with aSAH using a national HER database [10]. Despite achieving a high AUC in predicting mortality, the model’s performance was limited to patients who had received vasopressors.

Hence, there is a compelling imperative to advance the development of machine learning models capable of comprehensively assimilating the extensive clinical data within the EHR system, ensuring their adaptability across diverse data sources, and enabling the early prediction of mortality risk.

In this paper, we used a large national EHR database to build machine learning models for predicting mortality risk for aSAH patients. The resulting model can subsequently serve as a valuable tool for conducting real-time bedside mortality predictions following aSAH.

## Materials and methods

### Ethics statement

The study was approved by the Office of Research Support Committees at the University of Texas Health Science Center at Houston. The approval number is HSC-SPH-19-0976.

### Data source

Data for this study was extracted from the Cerner Health Facts EHR database, which comprised de-identified EHR data from over 700 hospitals and clinics in the United States. Cerner Health Facts EHR database included structured data such as patient demographics, diagnoses, procedures, lab results, medications, vital signs, and other clinical observations. We utilized EHR data that were collected between 2000 and 2018. We followed the “Guidelines for Developing and Reporting Machine Learning Predictive Models in Biomedical Research: A Multidisciplinary View” [18].

### Cohort definition and primary outcome

We included patients who were treated in hospital and diagnosed with SAH based on the International Classification of Diseases, Ninth Revision, Clinical Modification (ICD-9-CM) diagnosis code ICD-9-CM 430 and International Classification of Diseases, Tenth Revision, Clinical Modification (ICD-10-CM) I60.X. To maximize specificity, we curated aSAH patients by limiting data to only those patients with one of the following interventions: aneurysmal clipping (ICD-9-CM 39.51) or coiling (ICD-9-CM 39.72), cerebral angiogram (ICD-9-CM 88.41), or treatment with nimodipine.

To avoid the potential confounding effect of trauma, we excluded patients diagnosed with traumatic SAH (ICD-9-CM codes 800.0–804.9, 850.0–854.1, and 873.0–873.9, and ICD-10-CM codes S06.6X). We also excluded patients aged less than 18 years. The primary objective of this study was to predict the risk of in-hospital mortality for nontraumatic aSAH patients who stayed in hospital for at least 24 hours. We excluded patients who died within the 24 hours after hospital admission and patients with unknown gender and unknown race. Following these exclusions, the population size available for subsequent model analysis was reduced to 6,728 individuals. More details about the cohort definition can be found in S1 Appendix.

**Fig 1:**
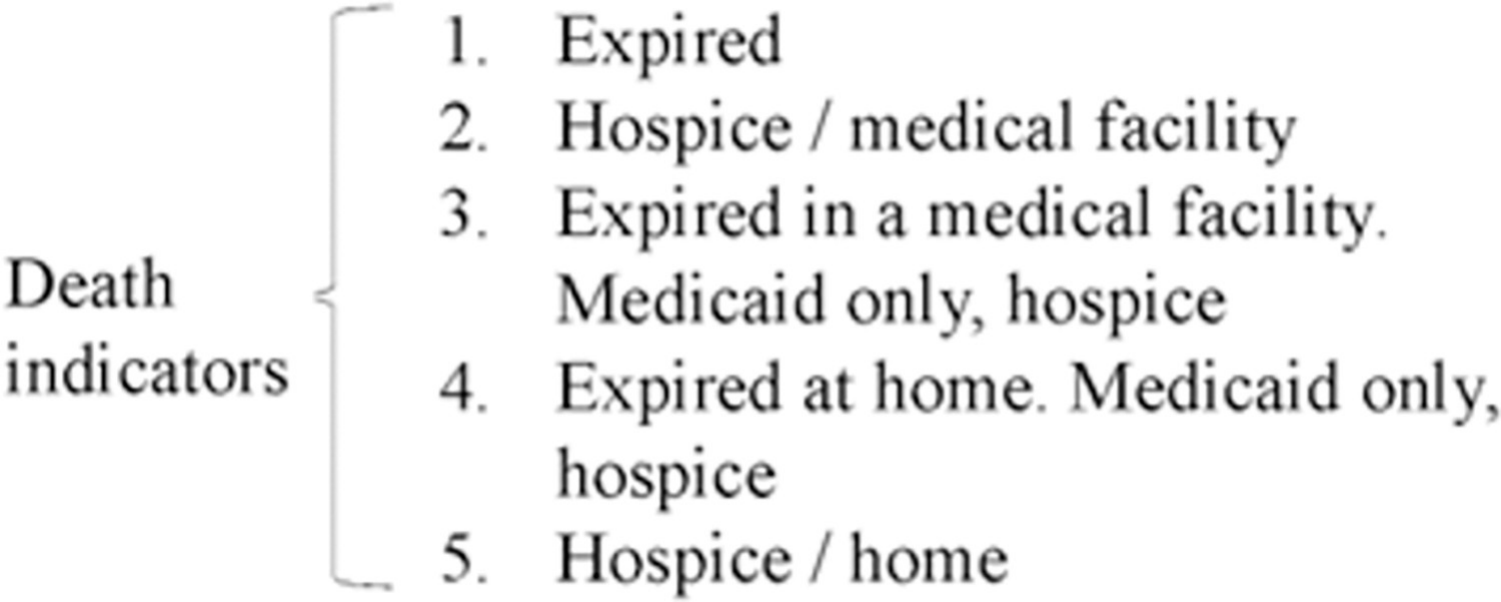
The definition of death from discharge dispositions.

In the Cerner database, the death status can be derived by the patient discharge disposition of an encounter. There were 24 valid categories (excluding the unknown categories) of patient discharge disposition in the cohort of aSAH patients. In this study, we considered patients who had one of the five categories of discharge disposition description in the following Fig 1 as dead cases. Subjects that had any other discharge dispositions were assumed to be alive and not imminently about to die at discharge.

## Results

### Basic characteristics

The cohort used for developing the prediction model consisted of 6,728 individuals. Among them, 1,000 patients (15%) experienced mortality. The median age of the cohort was 56 years, with an interquartile range of 47 to 66. The majority of patients were female, accounting for 63% of the cohort. The median length of stay in the hospital was 14 days. The Glasgow Coma Scale was documented for 2,638 patients (39%) within this cohort, with an average Glasgow Coma Scale of 12 (Table 1).

**Table 1:** Baseline characteristics of patients.

	Cohort size (6728)
Mortality, n (%)	1000 (15)
Female sex, n (%)	4257 (63)
Median LOS in days, mean (IQR)	14 (8–22)
Median age in years, mean (IQR)	56 (47–66)
Age	
<40, n (%)	832 (12)
40–49, n (%)	1265(19)
50–59, n (%)	1963 (29)
60–69, n (%)	1479 (22)
>70, n (%)	1189 (18)
Race	
White, n (%)	4793 (71)
African American, n (%)	1612 (24)
Other, n (%)	323 (5)
GCS^1^	
3–5, n (%)	270 (10)
6–8, n (%)	331 (13)
9–11, n (%)	171 (6)
12–14, n (%)	413(16)
15, n (%)	1452 (55)

IQR: interquartile range; LOS, hospital length of stay; GCS, Glasgow Coma Scale. 1 The GCS was available and analyzed for 995 (40%) patients

### Machine learning performance

The potential predictors considered in this study encompassed a wide range of clinical information, all collected within the initial 24 hours following hospital admission. These predictors included baseline demographic variables (such as age, gender, and race), binary procedure codes, binary medication data, binary diagnosis codes, and baseline values of laboratory tests and clinical observations. For a more comprehensive understanding of the data processing procedures, readers are encouraged to consult the relevant reference [19]. The missing data from lab tests and clinical observations were imputed with MisForest which demonstrated robust performance compared to other commonly used imputation techniques [19,20]. Furthermore, all predictors were scaled by the MinMax scaler [21]. In total, there were 330 predictors including: 3 demographical, 48 procedural, 64 diagnostic, 149 medications, 39 lab tests, and 27 clinical events.

The cohort was randomly split into training (70%) and validation (30%) data sets. We employed logistic regression with the sure independence screening (SIS) as the model selection method [22]. Additionally, we evaluated the performance of various other machine learning methods for comparison, including Support Vector Machine (SVM) [23], random forest [24], gradient boosting machine (GBM) [23], and multilayer perceptron (MLP) [23], using the R package caret [25]. S1 Appendix provides more details about the machine learning methods. The distinctive advantage of the SIS method lies in its two-step approach: initially, it conducts univariate logistic regressions to filter out features with weak marginal correlation to the response variable. Subsequently, it employs logistic regressions with LASSO penalty to select significant variables from the subset identified in the first step. This approach, in contrast to other model selection methods like LASSO, yields a reduced number of significant predictors, enhancing the interpretability of the factors influencing the outcome. Tuning parameters for the machine learning methods were selected via 5-fold cross-validation. The AUC of those machine learning methods to predict mortality for aSAH is presented in Table 2, while additional metrics such as sensitivity and specificity are detailed in Table C in S1 Appendix.

**Table 2:** The AUCs of different machine learning methods to predict mortality.

Machine learning methods	AUC
Logistic (SIS)	0.806
SVM	0.805
Random forest	0.795
GBM	0.819
MLP	0.786

### Risk factors identified by SIS

**Fig 2:**
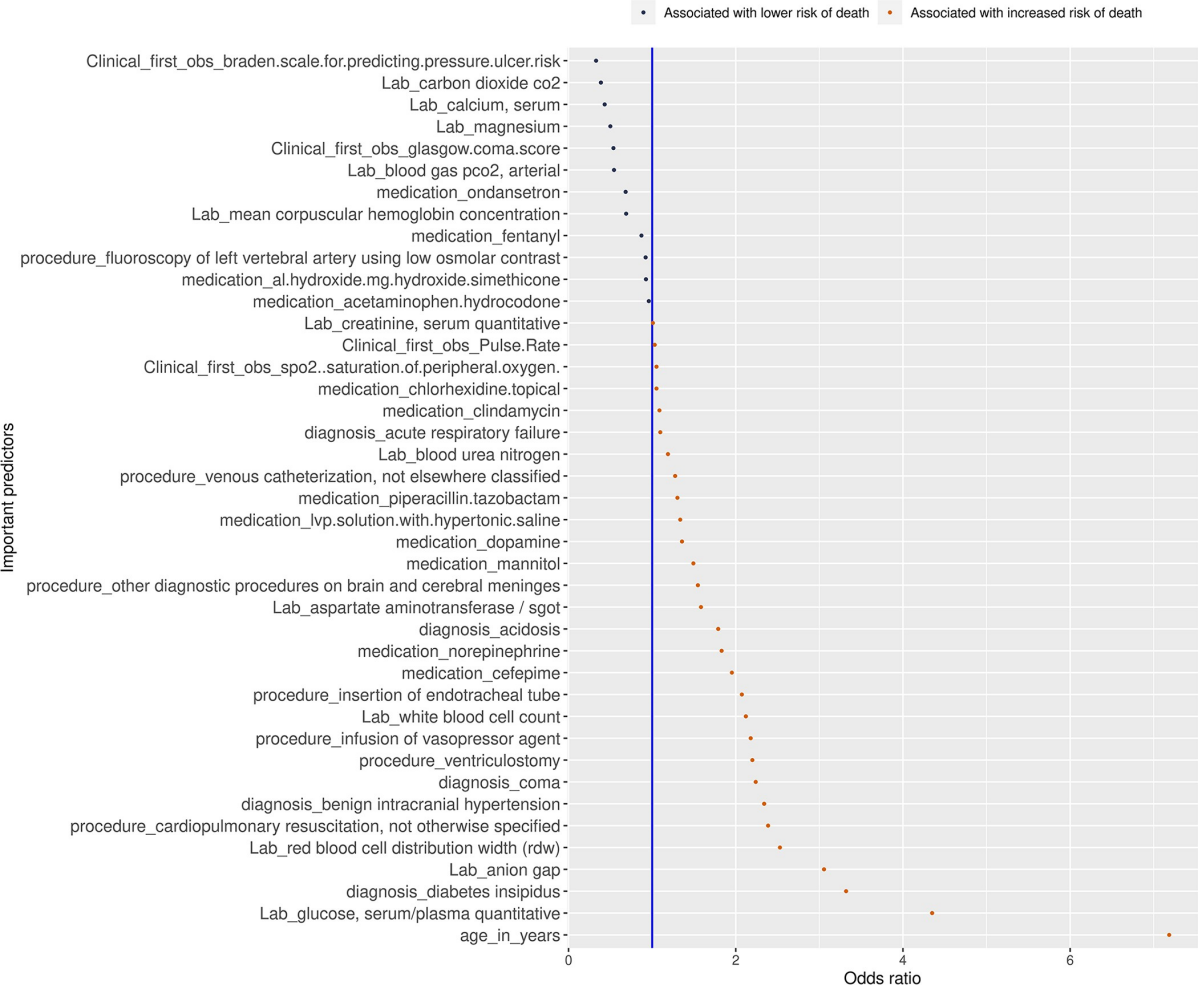
The predictors that are selected by the SIS prediction model and corresponding odds ratios.

While GBM exhibits superior performance over logistic regression with a notable 0.013 increase in the AUC, we must also underscore the significance of clinical interpretation in our analysis. Logistic regression, even while preserving a considerable portion of its predictive capability, offers the advantage of elucidating the impact of predictors on the outcome through metrics such as odds ratios. The final predictors selected by the logistic regression model with SIS were 42 of 330 predictors. See Fig 2 for the odd ratios and descriptions of the selected variables. The mean and standard deviation of the laboratory and clinical event features, as well as the count and percentage of medications, diagnoses, and procedures, separately for the death group and the non-death group are available in Table D and Table E in S1 Appendix.

In comparison to the findings reported in paper [10], our study involved a broader set of input predictors (330 as opposed to 185) due to the cohort restrictions in paper [10], which focused solely on patients treated with vasopressors. Our analysis revealed that 8 out of the 26 selected predictors in paper [10] were also identified by our model. These overlapping predictors include creatinine, anion gap, glucose, Braden scale for predicting pressure ulcer risk, fentanyl, Glasgow Coma Score, mannitol, and ondansetron. Clinically, the Braden scale and Glasgow Coma Score serve as valuable indicators of patient severity. Our analysis, as depicted in Fig 2, affirms that a higher Braden scale or Glasgow Coma Score is associated with a reduced risk of mortality, further corroborating the utility of these metrics in predicting patient outcomes [26].

Predictors that were selected in the first 24-hour scenario in reference [10], but were not chosen by our model, included osmolality, arteriography of cerebral arteries, clipping of aneurysm, acetaminophen, acetaminophen-oxycodone, aspirin, cefazolin, docusate, glucose, glycopyrrolate, labetalol, lidocaine, morphine, neostigmine, nimodipine, phenylephrine, propofol, and weight. we observed that phenylephrine and nimodipine were omitted from our model’s selection. This divergence might be attributed to the fact that the patients in our cohort were not specifically required to receive vasopressors. Consequently, the association between vasopressors (such as phenylephrine) and mortality may have been attenuated in our dataset. This underscores the importance of harnessing comprehensive clinical information to predict mortality in aSAH patients from EHR.

## Discussion

In our study, we meticulously established the aSAH cohort using data from the Cerner database. A distinguishing aspect of our approach, in contrast to existing sources like clinical trial data or EHR data pertaining to SAH, is that our aSAH cohort boasted a larger sample size and did not impose the limitation of being confined to patients receiving vasopressors. This distinction afforded machine learning models the advantage of utilizing comprehensive clinical information for the early prediction of mortality in aSAH patients. When assessing model performance based on AUC, Gradient Boosting Machine emerged as the top-performing method, although it outperformed logistic regression by only a marginal 0.013 increase in AUC. It is essential to emphasize the significance of clinical interpretation in our analysis. Through the application of logistic regression with the SIS model selection method, we successfully identified 42 risk factors that demonstrated associations with the mortality of SAH.

The novelty of our study was the application of machine learning on a large SAH cohort from a national EHR database. In the existing literature, the population size for SAH from EHR was usually less than 1000 patients [12,13,17], while our cohort size was 6728. This larger cohort size substantially enhances the generalizability of our findings. Our approach encompasses the diverse data originating from various hospitals, thereby accommodating the inherent heterogeneity within the data sources. Consequently, the risk factors we’ve pinpointed utilizing this extensive SAH cohort hold greater potential to yield insightful clinical implications, thereby contributing to a deeper understanding of this medical condition.

While some predictors were directly related to aSAH pathophysiology, affecting the adverse outcomes, other predictors were markers that correlate with the outcome. We observed that the Braden scale for predicting pressure ulcer risk and the Glasgow coma scale are two particularly relevant variables that are associated with a lower risk of death following aSAH. This finding aligns with existing literature [17] and is likely attributed to the fact that a lower Braden or Glasgow Coma Scale score is indicative of a more severe aSAH rupture. Other variables that are associated with lower risk of death included the medications for symptomatic therapy such as ondansetron for nausea, fentanyl and acetaminophen-hydrocodone for pain control, and aluminum-magnesium hydroxide simethicone, an antacid. While these variables may not have a direct connection to the pathophysiology of aSAH itself, they are indirectly related to the severity of symptoms and the neurological status of the patients.

Our data further highlighted that age exhibited the most robust correlation with an increased risk of mortality following an aSAH, a finding consistent with observations from a previous study [17]. This is clinically unsurprising, as advancing age is associated with a decline in the body’s regenerative capabilities, leading to an increased susceptibility to complications. Another variable associated with an elevated risk of death was serum glucose. Hyperglycemia is a well-known predictor for poor outcomes after an aSAH. The underlying mechanism of hyperglycemia is primarily linked to the activation of the hypothalamic-pituitary-adrenal axis and the sympathetic autonomic nervous system. This activation results in elevated levels of stress hormones like catecholamines and cortisol, ultimately leading to increased serum glucose levels. Hyperglycemia contributes to an amplified systemic inflammatory response, heightened vasoconstriction, and increased coagulation, thereby raising the risk of delayed cerebral ischemia (DCI) and cerebral infarction. These adverse clinical outcomes, in turn, elevate the risk of death following an aSAH. Furthermore, our data indicated that the use of certain medications, such as piperacillin-tazobactam and cefepime (broad-spectrum antibiotics commonly employed in sepsis treatment), was associated with poor outcomes. While the use of these antibiotics is not directly linked to the pathophysiology of aSAH, it does reflect the severity of the patient’s illness. Similarly, the utilization of vasopressors like norepinephrine and dopamine was linked to an increased risk of death in our data, corroborating findings from the referenced paper [11]. These associations underscore the clinical significance of these variables in predicting outcomes following aSAH

Our study had certain limitations that should be acknowledged. Firstly, the inherent data quality issues associated with Electronic Health Records (EHR) included substantial missing data for clinical variables, inaccuracies in diagnoses, and errors in other clinical variable information. A further limitation pertains to the interpretability of machine learning methods. While we utilized the logistic regression model to offer some interpretable results, the variables selected by the model may not always align perfectly with established clinical knowledge. This disparity can pose challenges in drawing straightforward clinical inferences from the model’s findings.

## Supporting information

S1 Appendix Fig A. Patient count of different combinations of first encounter’s SAH ICD code groups. Table A. Code groups and their descriptions. Table B. Combined ICD code groups and their descriptions. Table C. Sensitivity, specificity, accuracy, negative predictive value, and positive predictive value under the optimal cutoff point by maximizing Youden Index. Table D. The mean and standard deviation of clinical events, labs and demographics in the selected predictors for the death group and non-death group. Table E. The count and percentage of the diagnoses, medications and procedures in the selected predictors for the death group and non-death group.
(DOCX)
