# Supplementary material for: Machine learning to predict mortality for aneurysmal subarachnoid hemorrhage (aSAH) using a large nationwide EHR database
Source: PLOS Digit Health. 2023 Dec 6;2(12):e0000400. doi: 10.1371/journal.pdig.0000400 (PMC10699620; doi:10.1371/journal.pdig.0000400)
Supplement: S1 Appendix — Fig A. Patient count of different combinations of first encounter’s SAH ICD code groups. Table A. Code groups and their descriptions. Table B. Combined ICD code groups and their descriptions. Table C. Sensitivity, specificity, accuracy, negative predictive value, and positive predictive value under the optimal cutoff point by maximizing Youden Index. Table D. The mean and standard deviation of clinical events, labs and demographics in the selected predictors for the death group and non-death group. Table E. The count and percentage of the diagnoses, medications and procedures in the selected predictors for the death group and non-death group. (DOCX) [file pdig.0000400.s001.docx]

# Appendix

## Details of cohort definition

Although some literature discussed and listed ICD codes for SAH[27], in order to contain as many ICD codes for our project as possible, we conducted keyword searches against the diagnosis description in the EHR database. Eventually, 192 unique ICD9/10 codes for SAH were collected. The unique ICD codes were then categorized into six code groups based on causes, symptoms and characteristics. See Table A.

Table A. Code groups and their descriptions

| ICD codes | Description |
| --- | --- |
| 800.X-804.X | fracture of skull with subarachnoid, subdural, and extradural hemorrhage |
| 430/I60.X | Nontraumatic subarachnoid hemorrhage |
| P52.5/P10.3/772.2 | Baby related subarachnoid hemorrhage |
| 852.0-852.2 | Subarachnoid hemorrhage following injury |
| S06.6X | Traumatic subarachnoid hemorrhage |
| I69.0X | Sequelae following nontraumatic subarachnoid hemorrhage |

We classified ICD code groups ‘800.X-804.X’ and ‘852.0-852.2’ as traumatic subarachnoid hemorrhage and deleted patients diagnosed with ‘P52.5/P10.3/772.2’ because those codes are related to babies. So, the filtered ICD code groups and their descriptions are shown in Table B.

Table B. Combined ICD code groups and their descriptions

| ICD codes | Description |
| --- | --- |
| 800.X-804.X/ S06.6X/852.0-852.2 | Traumatic subarachnoid hemorrhage |
| 430/I60.X | Nontraumatic subarachnoid hemorrhage |
| I69.0X | Sequelae following nontraumatic subarachnoid hemorrhage |

1. **SAH patient count based on the first SAH encounter**

We based on the first SAH encounter for our analysis. See Fig A for the number of patients whose first SAH diagnosis fell into different combinations of SAH ICD code groups. Traumatic SAH and nontraumatic SAH presented different clinical symptoms and complications. Treating the two types of SAH as the same disease would generate biased results in subsequent analyses. In our further cohort characterization, we restricted our study on patients whose first SAH diagnosis was nontraumatic SAH. Since we were only interested in patients admitted to hospital with SAH but not sequelae following SAH, we dropped the patients with the sole diagnosis code of sequelae following nontraumatic subarachnoid hemorrhage. We treated patients with both diagnosis code group of sequelae following nontraumatic SAH and the diagnosis code group of nontraumatic SAH as nontraumatic SAH patients. After excluding patients less than 18 years old, the cohort size for the first encounter based nontraumatic SAH patients was 28295.


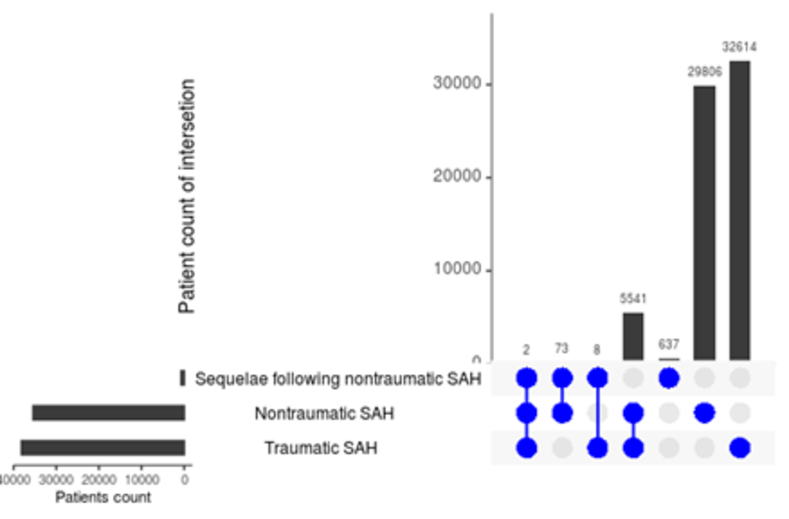


Fig A. Patient count of different combinations of first encounter’s SAH ICD code groups

1. **Define aSAH**

Since the occurrence of aSAH makes up over 80% of SAH and the pathological difference between arteriovenous malformation and aSAH, we concentrated our study on aSAH. In order to accurately identify aSAH patients from the administrative database, we required each predefined SAH patient to be inpatient and with at least one record of clipping, coiling, nimodipine, or cerebral angiogram. The final cohort population of aSAH was 7,492 (before excluding patients who died within the 24 hours after hospital admission and patients with unknown gender and unknown race ).

## Machine learning methods

- - 1. **Logistic regression with SIS**

To solve the high dimension variable problem, one way is to use shrinkage methods such as LASSO and its variations. Another method for dealing with this issue, which is especially suitable for dealing with ultrahigh dimensional problems, is SIS [22]. For a linear regression model

$$\boldsymbol{y}=\boldsymbol{X\beta}+\boldsymbol{\epsilon,}$$

suppose $\boldsymbol{y}=\left( y_{1}, \ldots, y_{n} \right)$ is the response vector with dimension $n$, $\boldsymbol{X}=\left( \boldsymbol{x}_{\left( 1 \right)}, \ldots, \boldsymbol{x}_{\left( p \right)} \right)$ is the $n\times p$design matrix, $\boldsymbol{x}_{\left( i \right)}$ is the $i$th covariate among the total $p$ covariates; $\boldsymbol{\beta}=\left( \beta_{1}, \ldots,\beta_{p} \right)^{T}$ is the $p$-dimensional regression coefficient vector, and $\boldsymbol{\epsilon}=\left( \epsilon_{1},\ldots, \epsilon_{p} \right)^{\boldsymbol{T}}$ is the $n$-dimensional error vector. SIS is performed in two different steps. In the first step, it assumes a given number of covariates that influence the outcome while the rest are considered as noise. Let $\hat{\mathcal{M}}$represent those significant variables. Then, based on the magnitude of the marginal correlation $\hat{corr}\left( \boldsymbol{x}_{\left( i \right)}, \boldsymbol{y} \right)$ of $\boldsymbol{x}_{\left( i \right)}$ and $\boldsymbol{y}$, SIS chooses the top $d$ covariates to build the set $\hat{\mathcal{M}}$; in other words,

$$\hat{\mathcal{M}}=\left\{ 1\leq i\leq p:\left| \hat{corr}\left( \boldsymbol{x}_{\left( i \right)}, \boldsymbol{y} \right) \right| is among the top d largest ones \right\},$$

where  $\hat{corr}$ stands for the sample Pearson correlation.

Next, common regularization variable selection methods including Ridge regression, LASSO, and its various extensions, can be utilized to further screen the reduced set $\hat{\mathcal{M}}$. Here, we present logistic regression with the elastic net penalty based on the covariates in ${\hat{\mathcal{M}}}$.

Logistic regression is the most widely used model for binary outcome data. Mathematically, it takes the form

$$\begin{aligned} \log\left( \frac{\pi\left( \boldsymbol{x}_{i} \right)}{1-{\pi(\boldsymbol{x}}_{i})} \right)=\boldsymbol{x}_{i}^{T}\boldsymbol{\beta},\# \end{aligned}$$

where $\pi\left( \boldsymbol{x}_{i} \right)=E\left( y_{i} \right)=P\left( y_{i}=1 | X=\boldsymbol{x}_{i} \right)=1-P\left( Y=0 | X=\boldsymbol{x}_{i} \right)$. $y_{i}$ is the binary outcome and $\boldsymbol{x}_{i}$ are the covariates in $\mathcal{M}_{*}$. In our example, $y_{i}$ is the death status of patient $i$ and $\boldsymbol{x}_{i}$are some clinical variables of patient $i$. The equation above implies

$$\pi\left( \boldsymbol{x}_{i} \right)=\frac{\exp\left( \boldsymbol{x}_{i}^{T}\boldsymbol{\beta} \right)}{1+\exp\left( \boldsymbol{x}_{i}^{T}\boldsymbol{\beta} \right)}.$$

from which we can infer the loglikelihood of logistic regression. The maximum likelihood estimate for $\boldsymbol{\beta}$ in the logistic regression with the elastic net penalty is

$$\min_{\boldsymbol{\beta}} n^{-1}\sum_{i=1}^{n} \left( -y_{i}{(\boldsymbol{x}}_{i}^{T}\boldsymbol{\beta})+log(1+exp(\mu+\boldsymbol{x}_{i}^{T}\boldsymbol{\beta})) \right)+\lambda_{1}\left\| \boldsymbol{\beta} \right\|_{1}+\lambda_{2}\left\| \boldsymbol{\beta} \right\|^{2}.$$

The elastic net penalty term $\lambda_{1}\left\| \boldsymbol{\beta} \right\|_{1}+\lambda_{2}\left\| \boldsymbol{\beta} \right\|^{2}$ is a combination of the $L_{1}$ and $L_{2}$ penalties of the lasso and ridge methods both of which are variable selection methods. Adding the elastic net into logistic regression can help reduce the variable dimensions and automatically select important variables.

- - 1. **Support vector machine (SVM)**

SVM is one of the most robust prediction machine learning methods and is widely used in classification and regression[28]. SVM aims to find a separating hyperplane that maximizes the distance of the closest points to the margin. Let $x\in R^{N}$ and $y\in R$. Define a hyperplane by

{$x:f\left( x \right)=\beta_{0}+{h(x)}^{T}\beta$ = 0}

where $h(x)$ denotes some nonlinear mapping of $x$. The inner product of $h(x)$, $K\left( x,x^{'} \right)= \left\langle h\left( x \right), h(x^{'}) \right\rangle$, could be replaced by varying kernel functions as follows:

dth-Degree polynomial: $K\left( x,x^{'} \right)={(1+\left\langle x, x^{'} \right\rangle)}^{d}$,

Radial basis: $K\left( x,x^{'} \right)={exp(-\gamma\left\| x-x^{'} \right\|}^{2})$,

Neural network: $K\left( x,x^{'} \right)=tanh(\kappa_{1}\left\langle x, x^{'} \right\rangle+\kappa_{2})$.

The SVM model can be treated as one solving the optimization problem

$$\min_{\beta_{0},\beta} \sum_{i=1}^{N} {[1-y_{i}f(x_{i})]}_{+}+\frac{\lambda}{2}\left\| \beta\right\|^{2},$$

where the subscript “+” indicates the positive part.

In the R package Caret, Radial kernel is used as the default kernel of SVM.

- - 1. **Random forest**

Random forest is a tree-based regression and classification method[24]. It grows many trees, and in the end uses all trees to make the decision. There are two types of randomness in random forest which generate different trees and reduce the prediction variance. The first randomness is to select a subsample of the data each time. The second randomness is considering only a random subset of variables for splitting. After growing the tress $B$ times, the random forest regression predictor becomes

$$\hat{f}^{B}\left( x \right)=\frac{1}{B}\sum_{b=1}^{B} T(x;\Theta_{b})$$

where $\Theta_{b}$characterizes the $b$th random forest tree.

- - 1. **Gradient boosting machine (GBM)**

Boosting is a procedure that combines the ‘weak’ classifiers to produce a powerful classifier. It sequentially modifies the weight of prediction models, thereby generating a sequence of models $G_{m}\left( x \right), m=1,2,\ldots, M.$ The prediction of a two-class problem, with the outcome labeled as $Y\in\{-1,1\}$, is obtained by weighted average vote:

$$G\left( x \right)=sign(\sum_{m=1}^{M} \alpha_{m}G_{m}(x))$$

where $\alpha_{1}, \alpha_{2},\ldots\alpha_{M}$ denotes the weight of the contribution from each respective $G_{m}(x)$, $G_{m}(x)\in\{-1,1\}$. The algorithm AdaBoost.M1can be used to solve the weight and output the final prediction. For more details, readers can refer to Chapter 10 in [23].

- - 1. **Multilayer perceptron (MLP)**

Multilayer perceptron (MLP), also known as artificial neural networks (ANNs), is one of the basic classifiers in deep learning. MLP is a feedforward neural network where the values from prior layers directly move into the next layer. An ANN with a single hidden layer can be written as:

$$h_{ij}= \sigma_{1}\left( W_{j}^{'}X_{i}+b_{j} \right)$$

$$P\left( Y_{i}=1 \right)= \sigma_{2}(W_{0}^{'}H_{i}+ b_{0})$$

where $W_{j}$is a $p\times$1weight vector; $X_{i}$is the i-th observation, $b_{j}$ is a scalar, j = 1, 2, $\cdots$, m. $H_{i}$ is the single hidden layer for i-th observation, $H_{i}$ = ($h_{i1,}h_{i2}, \cdots h_{im}$)'. $W_{0}$ is a $m\times$1weight vector; and $b_{0}$ is a scalar. $\sigma_{1}$and $\sigma_{2}\mathrm{are}$ the active function, such as logistic sigmoid function, tanh function. $P\left( Y_{i}=1 \right)$ denotes the probability of the outcome being present for the $i$th sample. The parameters can be solved using backpropagation.

Within the R package caret, the function "mlp" sourced from RSNNS package is employed. The default settings encompass the utilization of standard backpropagation as the learning method, with a learning rate of 0.2. In our scenario, the hidden layer count spans from 1 to 5, and the epoch count is fixed at 100. The most proficient hidden layer configuration, determined through cross-validation, is utilized for the ultimate predictions.

## Evaluation metrics for the machine learning methods

Table C. Sensitivity, specificity, accuracy, negative predictive value, and positive predictive value under the optimal cutoff point by maximizing Youden Index

| methods | Logistic (SIS) | SVM | random forrest | GBM | MLP |
| --- | --- | --- | --- | --- | --- |
| optimal J | 0.15 | 0.18 | 0.17 | 0.09 | 0.08 |
| sensitivity | 0.74 | 0.65 | 0.77 | 0.82 | 0.77 |
| specificity | 0.75 | 0.82 | 0.72 | 0.7 | 0.69 |
| accuracy | 0.75 | 0.8 | 0.73 | 0.72 | 0.7 |
| npv | 0.94 | 0.93 | 0.95 | 0.96 | 0.94 |
| ppv | 0.34 | 0.39 | 0.32 | 0.33 | 0.3 |

Optimal J: the optimal cutoff point by maximizing Youden Index, which is defined as $J= {max}_{i}({sensitivity}_{i}+{specificity}_{i}-1)$.

NPV: negative predictive value.

PPV: positive predictive value

## Details of selected predictors by SIS

Table D. The mean and standard deviation of clinical events, labs and demographics in the selected predictors for the death group and non-death group

| group | OR | Predictors | non death group | death group |
| --- | --- | --- | --- | --- |
| clinical event | 1.052 | spo2..saturation.of.peripheral.oxygen. (%) | 97.96 (1.44) | 98.07 (1.38) |
| clinical event | 1.031 | pulse.rate (Beats per minute) | 83.80 (10.50) | 85.73 (11.60) |
| clinical event | 0.537 | glasgow.coma.score | 13.68 (2.51) | 11.95 (4.09) |
| clinical event | 0.329 | braden.scale.for.predicting.pressure.ulcer.risk | 17.23 (2.13) | 15.90 (2.80) |
| demographics | 7.185 | age_in_years (years) | 55.06 (13.87) | 61.86 (15.10) |
| lab | 4.349 | glucose, serum/plasma quantitative (mg/dL) | 141.80 (41.81) | 161.75 (53.45) |
| lab | 3.056 | anion gap (mmol/L) | 10.43 (2.27) | 11.19 (2.66) |
| lab | 2.529 | red blood cell distribution width (rdw) (%) | 13.43 (1.15) | 13.76 (1.66) |
| lab | 2.121 | white blood cell count (10*3/uL) | 12.17 (2.50) | 13.04 (3.51) |
| lab | 1.584 | aspartate aminotransferase / sgot (U/L) | 31.89 (21.87) | 41.33 (44.68) |
| lab | 1.189 | blood urea nitrogen (mg/dL) | 14.34 (5.40) | 15.86 (7.69) |
| lab | 1.009 | creatinine, serum quantitative (mg/dL) | 0.85 (0.53) | 0.94 (0.70) |
| lab | 0.689 | mean corpuscular hemoglobin concentration (g/dL) | 33.84 (0.78) | 33.69 (0.92) |
| lab | 0.544 | blood gas pco2, arterial (mm[Hg]) | 38.97 (4.04) | 38.18 (6.03) |
| lab | 0.5 | magnesium (mg/dL) | 1.95 (0.16) | 1.92 (0.22) |
| lab | 0.432 | calcium, serum (mg/dL) | 8.97 (0.46) | 8.84 (0.51) |
| lab | 0.387 | carbon dioxide co2 (mmol/L) | 25.02 (2.05) | 24.12 (2.69) |

Table E. The count and percentage of the diagnoses, medications and procedures in the selected predictors for the death group and non-death group

| group | OR | Predictors | non death group | death group |
| --- | --- | --- | --- | --- |
| diagnosis | 3.32 | diabetes insipidus | 39 ( 0.7) | 28 ( 2.8) |
| diagnosis | 2.34 | benign intracranial hypertension | 68 ( 1.2) | 37 ( 3.7) |
| diagnosis | 2.239 | coma | 13 ( 0.2) | 20 ( 2.0) |
| diagnosis | 1.79 | acidosis | 107 ( 1.9) | 64 ( 6.4) |
| diagnosis | 1.097 | acute respiratory failure | 99 ( 1.7) | 51 ( 5.1) |
| medication | 1.954 | cefepime | 15 ( 0.3) | 14 ( 1.4) |
| medication | 1.831 | norepinephrine | 103 ( 1.8) | 86 ( 8.6) |
| medication | 1.493 | mannitol | 428 ( 7.5) | 183 (18.3) |
| medication | 1.357 | dopamine | 92 ( 1.6) | 58 ( 5.8) |
| medication | 1.336 | lvp.solution.with.hypertonic.saline | 137 ( 2.4) | 78 ( 7.8) |
| medication | 1.302 | piperacillin.tazobactam | 72 ( 1.3) | 38 ( 3.8) |
| medication | 1.087 | clindamycin | 33 ( 0.6) | 14 ( 1.4) |
| medication | 1.052 | chlorhexidine.topical | 220 ( 3.8) | 93 ( 9.3) |
| medication | 0.96 | acetaminophen.hydrocodone | 563 ( 9.8) | 29 ( 2.9) |
| medication | 0.926 | al.hydroxide.mg.hydroxide.simethicone | 147 ( 2.6) | 13 ( 1.3) |
| medication | 0.872 | fentanyl | 2692 (47.0) | 367 (36.7) |
| medication | 0.683 | ondansetron | 3664 (64.0) | 493 (49.3) |
| procedure | 2.387 | cardiopulmonary resuscitation, not otherwise specified | 4 ( 0.1) | 13 ( 1.3) |
| procedure | 2.199 | ventriculostomy | 339 ( 5.9) | 146 (14.6) |
| procedure | 2.179 | infusion of vasopressor agent | 55 ( 1.0) | 51 ( 5.1) |
| procedure | 2.073 | insertion of endotracheal tube | 398 ( 6.9) | 241 (24.1) |
| procedure | 1.547 | other diagnostic procedures on brain and cerebral meninges | 19 ( 0.3) | 10 ( 1.0) |
| procedure | 1.275 | venous catheterization, not elsewhere classified | 377 ( 6.6) | 149 (14.9) |
| procedure | 0.922 | fluoroscopy of left vertebral artery using low osmolar contrast | 96 ( 1.7) | 10 ( 1.0) |
